# Supplementary material for: Nano-Topographically Guided, Biomineralized, 3D-Printed Polycaprolactone Scaffolds with Urine-Derived Stem Cells for Promoting Bone Regeneration
Source: Pharmaceutics. 2024 Jan 31;16(2):204. doi: 10.3390/pharmaceutics16020204 (PMC10892771; doi:10.3390/pharmaceutics16020204)
Supplement: Supplementary file 1 [file pharmaceutics-16-00204-s001.zip › pharmaceutics-2747654-supplementary.pdf]

Supplementary Table S1. Primers for real-time polymerase chain reaction.

Target gene	Forward primer sequence (5-3)	Reverse primer sequence (5-3)
GAPDH	ACAACTTTGGTATCGTGGAAGG	GCCATCACGCCACAGTTTC
RUNX2	CCAACCCACGAATGCACTATC	TAGTGAGTGGTGGCGGACATAC
ALP	ACCACCACGAGAGTGAACCA	CGTTGTCTGAGTACCAGTCCC
COL1A1	GCCCAGAAGAACTGGTACATCAG	CGCCATACTCGAACTGGAATC
OCN	CCCCCTCTAGCCTAGGACC	ACCAGGTAATGCCAGTTTGC
